# Coplanar embedding of multiple 3D cell models in hydrogel towards high-throughput micro-histology

**DOI:** 10.1038/s41598-022-13987-4

**Published:** 2022-06-15

**Authors:** Sarah Heub, Fatemeh Navaee, Daniel Migliozzi, Diane Ledroit, Stéphanie Boder-Pasche, Jonas Goldowsky, Emilie Vuille-Dit-Bille, Joëlle Hofer, Carine Gaiser, Vincent Revol, Laura Suter-Dick, Gilles Weder

**Affiliations:** 1 CSEM SA, Jaquet-Droz 1, 2002 Neuchâtel, Switzerland; 2 School of Life Sciences, University of Applied Sciences and Arts Northwestern Switzerland, 4132 Muttenz, Switzerland; 3 Swiss Centre for Applied Human Toxicology (SCAHT), 4001 Basel, Switzerland

**Keywords:** High-throughput screening, Experimental models of disease, Cancer models, Toxicology

## Abstract

Standardised and high-throughput methods have been developed for the production and experimental handling of some 3D in vitro models. However, adapted analytical tools are still missing for scientists and researchers to fully exploit the potential of complex cellular models in pre-clinical drug testing and precision medicine. Histology is the established, cost-effective and gold standard method for structural and functional tissue analysis. However, standard histological processes are challenging and costly to apply to 3D cell models, as their small size often leads to poor alignment of samples, which lowers analysis throughput. This body of work proposes a new approach: *HistoBrick* facilitates histological processing of spheroids and organoids by enabling gel embedding of 3D cell models with precise coplanar alignment, parallel to the sectioning plane, thus minimising the loss of sample material. HistoBrick’s features are compatible with automation standards, potentially allowing automated sample transfer from a multi-well plate to the gel device. Moreover, HistoBrick’s technology was validated by demonstrating the alignment of HepG2 cultured spheroids measuring 150–200 µm in diameter with a height precision of ± 80 µm. HistoBrick allows up to 96 samples to be studied across minimal sections, paving the way towards high-throughput micro-histology.

## Introduction

**Figure 1:**
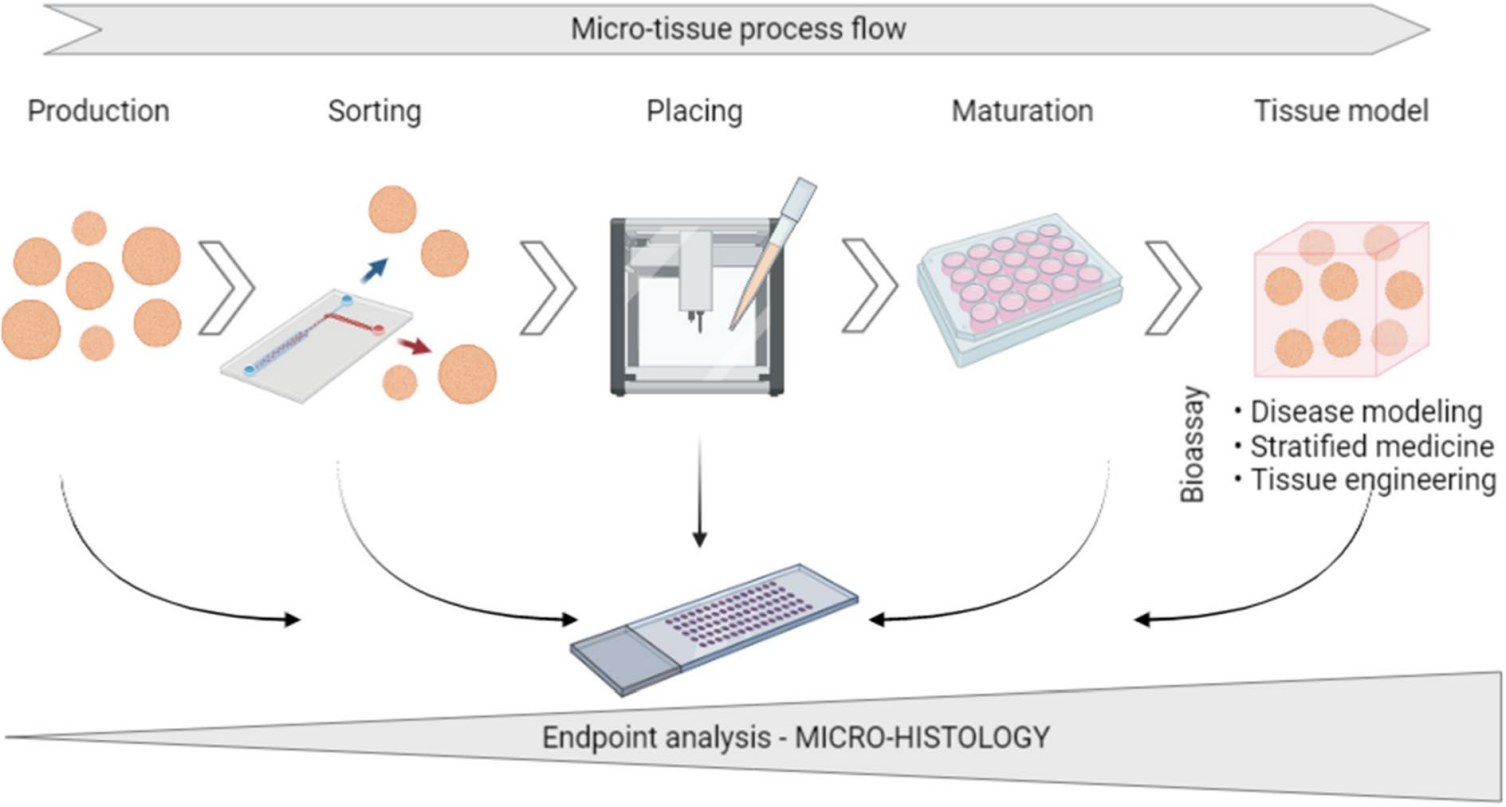
Micro-tissue processing chain showing the increasing need for micro-histology. In addition to endpoint analysis and by giving access to the micro-anatomy and biology of micro-tissues, micro-histology helps to determine the effect of processing steps on the samples (e.g., stress or damages affecting the morphology and biological functions, when combined with immunohistochemistry). Thus, it is the method of choice for quality control for the implementation of new methods serving the complete micro-tissue process flow, from production to tissue model development. Created with BioRender.com.

In vitro three-dimensional (3D) cell models have been gaining traction, as they enable better physiologically relevant tissue functions, architecture and interfaces compared to two-dimensional (2D) cultures. Patient-derived complex in vitro models reflect the unique genetic makeup. Moreover, omics and drug screening analysis are highly facilitated compared to experiments on animals^1^. In this context, complex in vitro models such as spheroids, organoids or tumouroids (collectively named micro-tissues), are being extensively used for disease modelling, pre-clinical drug development and tissue engineering^2^. Micro-tissues thereby support the implementation of personalised medicine^3^. Complex 3D cell models^4^ were primarily used to elucidate cell biology aspects^5^, while the development of tools and standardised methodologies for production, sorting, placing, maturation and analysis followed. As such, high-throughput analysis and quality control of complex cellular systems remain an ongoing challenge. Histology is the gold standard method for the analysis of the micro-anatomy of tissues; thus, 3D cell models' histological analysis is a logical consequence. Combined with immunohistochemistry, it provides information on tissue morphology and composition with the visualisation of specific proteins or antigens. Micro-histology is a powerful technique for quality control during all procedural steps of micro-tissue technology development with an increasing need toward endpoint analysis (Fig. 1).

However, current histological processes are not adapted to handle micro-tissues because they are smaller than biopsy samples and almost transparent. On top of this, technical handling issues can also lead to slow and cumbersome processing, producing results with a limited yield that cannot be reproduced using automation^6^.

Initially, histological processing requires that micro-tissues be embedded in a hydrogel, e.g., agarose, to facilitate their manipulation for subsequent embedding in paraffin^7,8^. When undertaking random embedding, dozens of micro-tissue replicates are needed to ensure enough suitable material is available for relevant histological analysis. This number of replicates seems unnecessarily large, and much of the material might go to waste considering that it could be more efficient to analyse fewer sample sections, especially as the accurate alignment of 5–10 micro-tissues in the same focal plane could provide sufficient information for robust analysis. There is the possibility to align multiple samples more efficiently in a plane, e.g., by centrifugation of micro-tissues in 0.5% agarose in a cryotube, which was alluded to in a recent study^9^. However, the pooling approach used in this study remains costly and still wastes valuable biological material.

Alternatively, manual transfer and positioning of micro-tissues is too time consuming and causes bottlenecks during automation. Instead, an elegant alternative to manual and random agarose embedding relies on parallelised hydrogel embedding using tissue microarrays (TMA)^7,8^. TMA work well to position samples in 2D arrangements, and they allow for the implementation of standard format methods used in cell culture, e.g., multi-well plates. With TMA, the traceability of each experimental condition can also be guaranteed and can be resultantly applied to 3D cell models^10,11^, and to the histology of small organism models, such as zebrafish larvae^12^. However, the random vertical positioning of samples within an obtained gel block makes it unlikely that all the micro-tissues will be located within the same section. Despite reducing the number of micro-tissues per experimental condition compared to pooling, TMA still requires many sections to be processed (e.g., stained and imaged) to obtain all the relevant data about the samples, resulting in high material costs and long time-to-results.

Several other studies have investigated different approaches with the aim of achieving high parallelisation of micro-tissue embedding for histological purposes (consisting of sectioning, staining and imaging). One method consisted of investigating up to 96 spheroids in parallel, using an agarose block designed with an array of manually filled cylindrical wells in order to analyse the impact of misalignment within the sectioning plane^8^. Another study used centrifugation through a funnel manifold to load micro-tissues into an agarose block with an array of cylindrical wells^13^. Nevertheless, it required manual manipulation of the plate to prepare the transfer. Other publications^14–16^ have proposed the formation and culture of the 3D cell models and their embedding on the same device. However, such labware is highly specific and incompatible with existing standard micro-tissue production platforms. To these authors’ knowledge, the coplanarity of the specimens within the gel block did not undergo investigation. Moreover, histological processing may affect the geometry of the agarose block and the relative position of the embedded samples.

**Figure 2:**
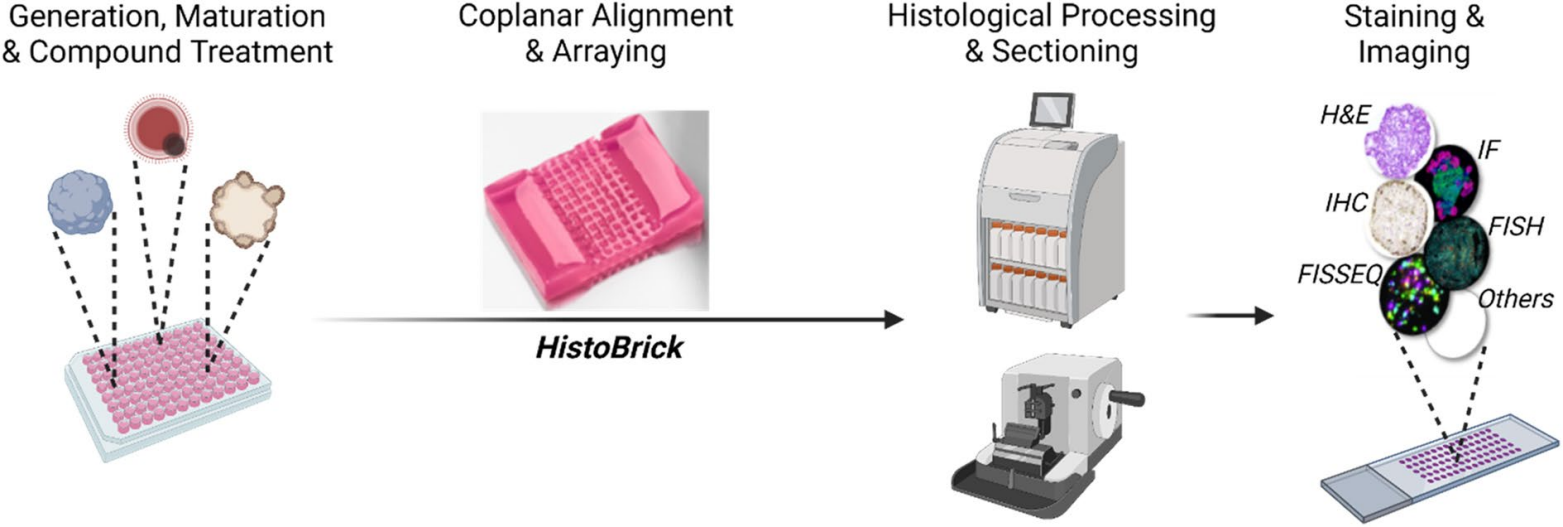
Schematic showing the pipeline of histological analysis on micro-tissues. HistoBrick provides the coplanar alignment and array arrangement needed to bridge 3D cell model production and histological processing towards high-throughput analysis of formalin-fixed and paraffin-embedded sections. Created with BioRender.com.

This paper proposes a cost-effective and efficient method for the high-throughput histological processing of micro-tissues. A user-friendly and robust method is described for the preparation of multiple samples in a standard array format using an agarose-based substrate: HistoBrick (Fig. 2). HistoBrick was designed to facilitate sample positioning, gel embedding, dehydration, paraffin embedding, sectioning and optical imaging whilst reducing the number of required sections in need of analysis by aligning the samples on a singular plane. The relative alignment of the specimens within HistoBrick and the positioning of the block on a microtome can be assessed. Moreover, HistoBrick’s approach is compatible with standard automation processes format and paves the way towards the standardisation and high-throughput automation of micro-histology.

## Methods

### Silicone mould fabrication

**Figure 3:**
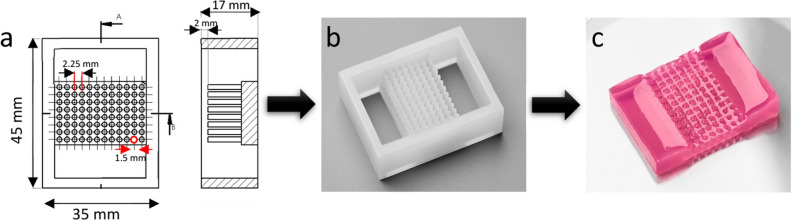
Histobrick design and hydrogel block. (**a**) CAD of the mould for the preparation of a 96-well HistoBrick, (**b**) Silicone mould fabricated by 3D printing, (**c**) Hydrogel-based HistoBrick containing 96 wells.

Two silicone moulds containing 96 vertical pillars were designed using SolidWorks (Dassault Systèmes), based on the configuration of the array pitch of commercial 1536 well plates. The pillars measure 1.5 mm in diameter and 15 mm in height. As shown in Fig. 3, the pillars were deliberately positioned in the middle of the mould, with two open spaces left available on either side, which once filled help to facilitate the handling of the hydrogel during moulding and unmoulding. The distance between the pillars and the bottom of the mould measures 2 mm in height, providing enough space to inject the hydrogel needed to obtain a HistoBrick. The mould designs were 3D printed with silicone A 50 at Spectroplast AG, Switzerland.

### HistoBrick preparation

**Figure 4:**
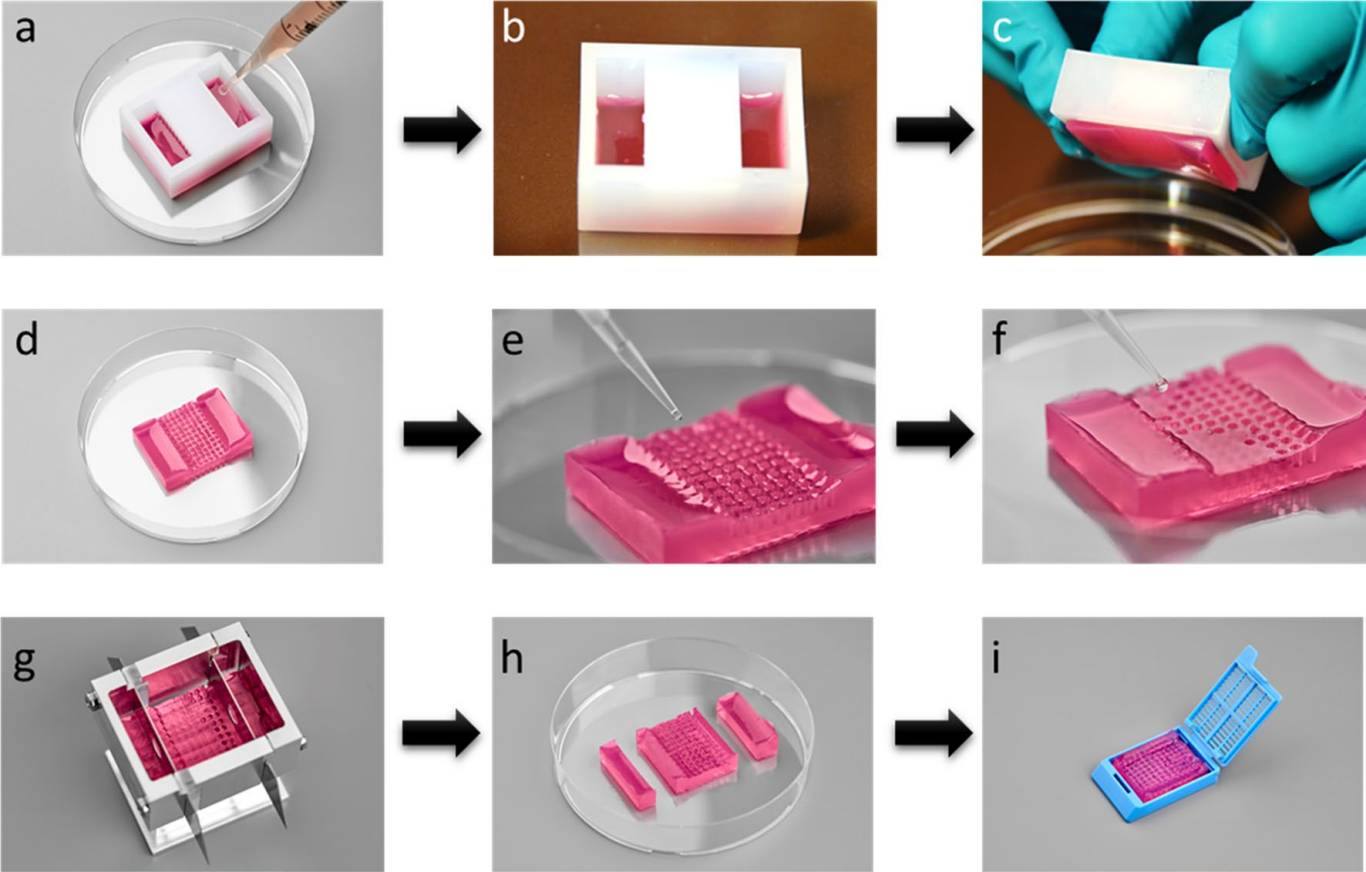
HistoBrick preparation and use. (**a**) Place the silicone mould in a glass petri dish with the pillars facing downwards and fill the mould with the agarose-HistoGel mixture from the side openings, (**b**) The filled mould should then be cooled at 4–5 °C for 2 h, (**c**) Unmold the HistoBrick by pressing the sides, (**d**) Unmolded HistoBrick, (**e**) Load fixed micro-tissues, (**f**) Add HistoGel in the wells and cool at 4–5 °C, (**g**) Cut the block using the slicing tool, (**h**) After dividing the HistoBrick into three segments, the two side segments can be discarded and the central segment containing the wells should be reserved for histological processing, (**i**) Place the central segment of HistoBrick in a histology cassette.

The main steps involved in the preparation of HistoBrick are depicted in Fig. 4a–d. Agarose (Sigma-Aldrich, A7174) was added to pre-warmed HistoGel (Epredia, HG-4000-012) in 1.5–2% w/v ratio, and the solution was stirred in an 80 °C water bath until the agarose had completely dissolved. The silicone mould was placed with the pillars facing downwards onto a petri dish; the assembly having been pre-warmed in an oven at 80 °C. Then 10 ml of the agarose-HistoGel mixture was gently poured into the heated silicone mould. These elements were then allowed to cool at room temperature for 10 min. Afterwards, the block was left to undergo gelation at 4 °C for 2 h to obtain sufficient mechanical stability for the unmolding. The solidified HistoBrick was released from the mould over a petri dish by applying gentle manual pressure in the middle area whilst holding both sides. The obtained HistoBrick was stored at 4 °C in deionised water and used within 14 days. To allow better visualisation of the wells before and after histological processing, additional HistoBrick samples were prepared by adding blue dye (Davidson Tissue Dye) to the agarose-HistoGel mixture.

### Micro-tissue positioning and hydrogel embedding

In this work, investigations were conducted using samples of human hepatocellular carcinoma (HepG2) (HB-8065, ATCC) spheroids (150–200 µm average diameter) generated using Sphericalplate 5D (SP5D, Kugelmeiers Ltd.). A detailed protocol for their preparation can be found in Supplemental Methods S1. The fixed HepG2 micro-tissues were manually transferred from an Eppendorf Tube to the HistoBrick, by pipetting one micro-tissue into each well using a single or multi-channel pipette (Fig. 4e). Pipette tips (20 µl) were pre-coated with 1% bovine serum albumin (BSA) (A3059, Sigma-Aldrich) to prevent adhesion of the micro-tissues. Each micro-tissue was aspirated together with approximately 5 µl of its aqueous buffer. The loaded tip was placed close to the bottom of the HistoBrick well without touching the gel, and the sample was dispensed slowly to prevent the trapping of air bubbles. The micro-tissues were allowed to settle and sediment in the well for 5 min. After transferring the micro-tissues, the mould was left to absorb the extra aqueous medium for approximately 1 h. With a new tip, 10–20 µl of HistoGel pre-warmed at 80 °C was added to every well (Fig. 4f). Lastly, the HistoBrick was left to cool for 1 h at 4 °C so the process of gelation could occur. It was then stored at 4 °C in deionised water and used within 3 days.

### Automated sample transfer from 96-well plate to HistoBrick

Several methods were developed and tested to investigate the transfer of HepG2 micro-tissues from an initial well plate to a HistoBrick, using a Microlab STAR Liquid Handling System (Hamilton) equipped with eight pipetting heads and Venus Software Version 2.1. Fixed HepG2 micro-tissues were placed (one per well) in a 96-well Corning Costar Ultra-Low Attachment (ULA) Multiple Well Plate (CLS7007, Merck) with 1 micro-tissue in 200 µl phosphate-buffered saline (PBS Gibco 2062235) in each well. Digital twin labware was created to support the HistoBrick, which was positioned on the deck using a dedicated holder. Transfer methods included the pre-coating of the tips with BSA.

### Histological processing: paraffin embedding, sectioning and staining

HistoBricks loaded with fixed HepG2 micro-tissues as previously described were trimmed to fit it into a histology cassette (Fig. 4g–i). A customised slicing tool was designed for this purpose as described in Supplemental Methods S2. The histological process comprised of a dehydration step followed by clearing and paraffin embedding, which were performed following standard laboratory procedures. The paraffin-embedded block was placed on a Leica RM2265 microtome for sectioning. In total 75 sections were obtained from one HistoBrick. The thickness of each slice was 4 µm. Every one out of three slices was stained with hematoxylin and eosin (H&E) following standard protocols after being mounted onto a glass slide.

### Optical imaging

Images of the stained sections were acquired with an Olympus VS120 whole slide scanner. Initially, an overview image of the entire glass slide was acquired at 4 × magnification to identify the areas containing micro-tissues. Afterwards, several micro-tissues distributed over the whole sample slice were selected and brought into focus to help correct tilting of the glass slide during the scanning process. Finally, a higher magnification (10 × or 20 ×) image of the whole section was acquired via scanning.

### Assessment of micro-well replication and shrinkage during histological processing

Blue dyed HistoBrick samples were prepared for better visualisation and their wells were filled with unstained HistoGel to assess their diameter at various stages (most notably on the freshly prepared HistoBrick and on slides obtained after histology processing). A bright-field image of the samples was then taken using an inverted microscope (Carl Zeiss Microscopy GmbH) and the diameters of the wells were measured based on manual circular fitting using the FIJI open-source platform^17^.

### Assessment of micro-tissue coplanarity

The images were processed in the FIJI platform^17^ and the TrackEM module^18^ was used to align the various sections’ well arrays. The images underwent two processes, firstly landmarks were manually selected on each image to guarantee the reliability of the alignment performance. Secondly, an automated rigid transformation of images was applied to the full stack to fasten the alignment. Subsequently, the location of the equator plane of each micro-tissue was identified visually as being the plane containing the largest section of the corresponding micro-tissue over the full image stack. When two consecutive sections of a micro-tissue were identified as being remarkably similar, their equator plane was chosen as being the one centred between the two sections. When several micro-tissues were embedded in the same well, since the locations of their equator plane was in close proximity (< 15 µm difference), they were averaged together. This procedure balanced the contribution of each well when performing fitting on the 3D spatial locations and ensured that the best fitting plane was unbiased by the wells containing additional micro-tissues. The 3D locations were then processed in MATLAB (2012, MathWorks) to identify the best fitting plane via mean-squared-error minimisation for each of the processed HistoBrick samples.

## Results

### Fabrication of HistoBrick

HistoBrick blocks composed of agarose can be easily produced using the dedicated silicone mould. The 96 wells within the HistoBrick measure 1.7 ± 0.1 mm in diameter after stabilisation in deionised water, resulting in a 6% deviation from the mould pillar diameter. However, the pitch of a standard 1,536 array is preserved, which would facilitate sample loading using an automated liquid handler.

### Compatibility with histological processing

**Figure 5:**
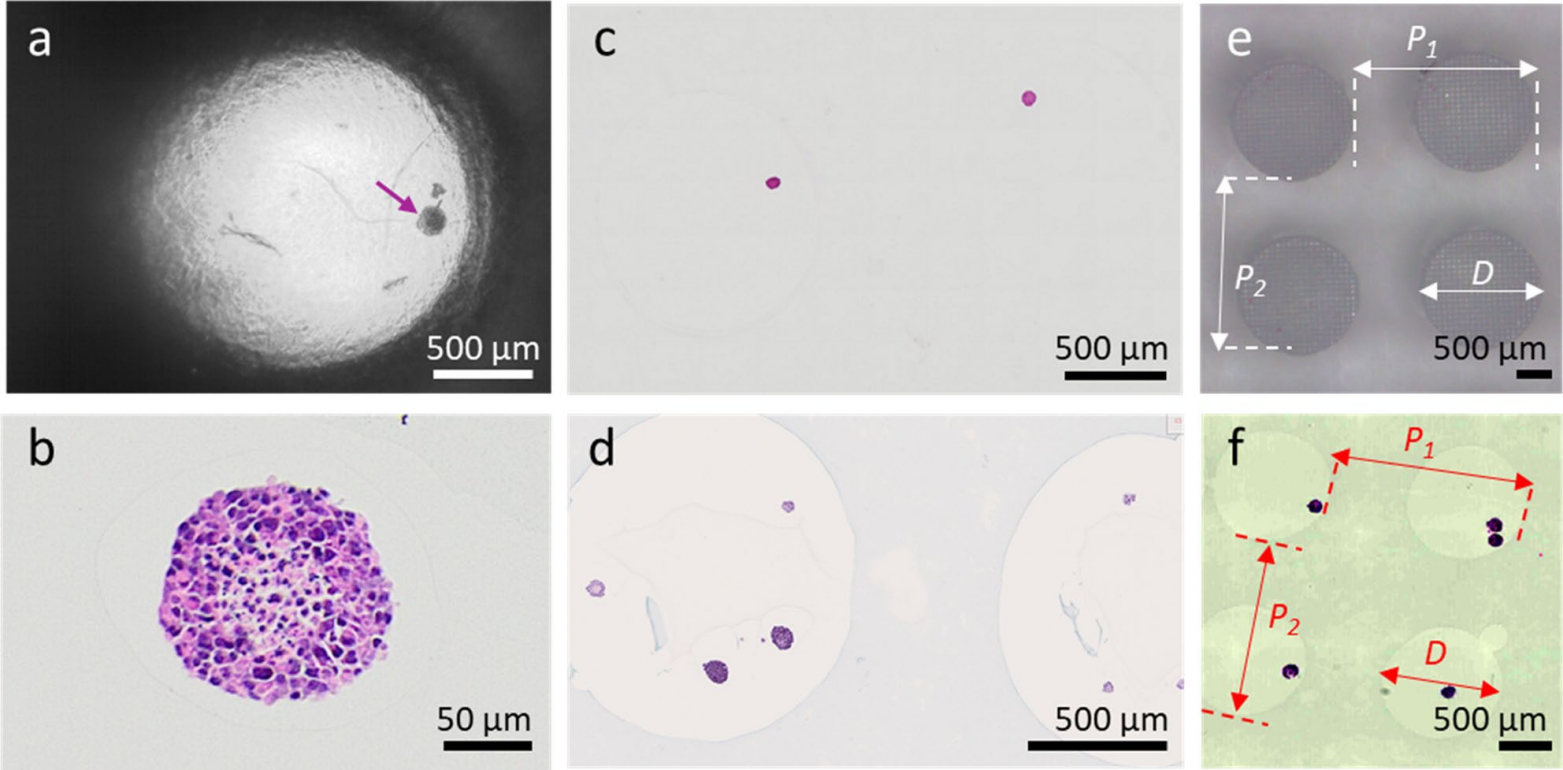
Images of HepG2 micro-tissues in a HistoBrick undergoing different processing steps. (**a**) A HepG2 micro-tissue (pointed by the arrow) after being embedded in the HistoBrick and topped with HistoGel. Note no air bubbles are trapped inside the micro-well, (**b**) H&E staining of a section of a single micro-tissue, (**c**) Microscopy image of a slice without dye. The well contours are difficult to determine. (**d**) Microscopy image of a slice using a blue coloured stain. Note the well contours are clearly visible. (**e**) Measuring the diameter and pitch of the wells in the HistoBrick before paraffin embedding, (**f**) Measuring the diameter and pitch of the wells in the HistoBrick after sectioning. The parameter D is the diameter whereas P_1_ and P_2_ are used to compare the pitch on the two axes of the micro-well array.

HistoBrick is suitable for histological processing steps including dehydration, paraffin embedding, sectioning with a microtome and staining. Comparing Fig. 5c and d shows the importance of having blue dye to facilitate the visualization of the wells. To determine the effect of the processing on the composite hydrogel block, the well diameters (D) and array pitch in two axes (P1 and P2) were measured on the slices of two blue coloured HistoBrick samples with embedded HepG2 micro-tissues (Fig. 5c–f). Results show a 34% shrinkage of the well diameter. The measured pitch after histology processing was 1.7 mm, corresponding to a 28% reduction. No air bubbles were observed after gel embedding or inside the histological sections (Fig. 5a). The HepG2 micro-tissues remained intact after H&E staining (Fig. 5b).

### HepG2 micro-tissue coplanarity after histology processing

**Figure 6:**
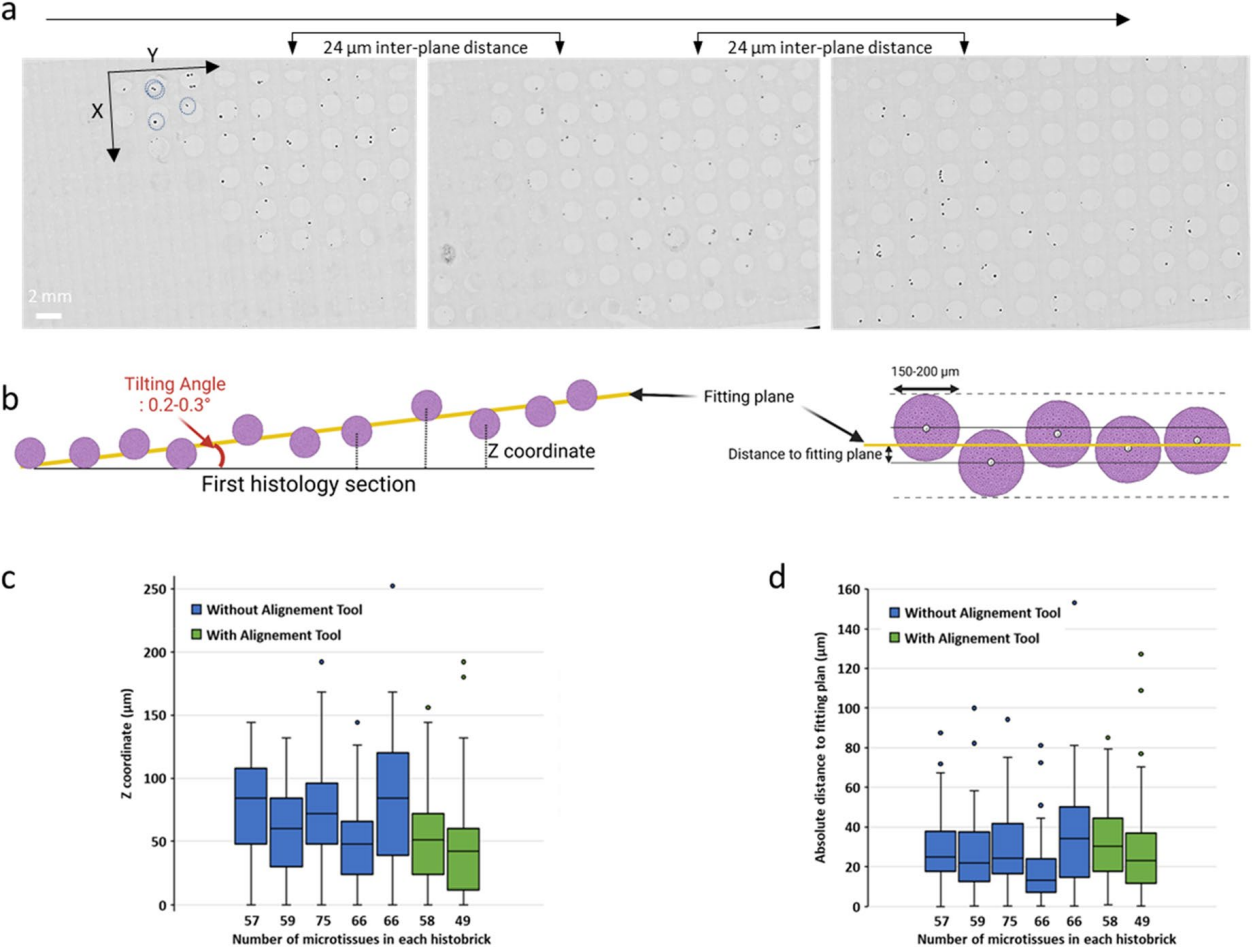
Coplanarity of embedded HepG2 micro-tissues. (**a**) Bright-field images of three H&E-stained slides from the same processed block. Light disks correspond to wells and dark dots correspond to micro-tissues, as in the example circled with dotted line, (**b**) Schematic of the position of the spheroids in the HistoBrick. Best fitting plane (yellow line) is an imaginary plane to compensate the tilting angle (in red, the angle formed by the best fitting plane with the plane of the microtome blade) which is the result of manual positioning of the block on the microtome, (**c**) Distance of micro-tissues’ centre from the first histology section for seven different HistoBrick samples [5 Histobricks were cut without alignment tool (blue boxes) and two HistoBricks were cut with alignment tool (green boxes)]. (**d**) Absolute distance of the micro-tissues’ centre from the best fitting plane for the same seven HistoBrick samples.

Since the throughput of histological analysis depends strongly on how the micro-tissues are positioned within the block during the sectioning procedure step, the authors of this paper characterised the alignment of HepG2 micro-tissues using the HistoBrick specialised approach. For this approach, the micro-tissue samples settle and sediment to the bottom of the wells, resulting in alignment on a horizontal plane. Figure 6a, shows three examples of bright-field images taken of the sections obtained for one 96-well HistoBrick sample after H&E staining. The HepG2 micro-tissues are easily identified as dark spots within the wells’ boundaries. As a result of the unprecise manual loading of HistoBrick from a sample suspension, the wells are sometimes empty or contain up to three micro-tissues. Figure 6b illustrates the positioning of the microtissues within a HistoBrick along the Z axis, relative to the first histology section and best fitting plane. Figure 6c shows the position of multiple microtissues from the first histology section in five HistoBrick blocks (in blue) and 2 other blocks prepared using the positioning tool (in green). Assuming that the histological process resulted in parallel surfaces of the paraffin and the original HistoBrick sample, the alignment plane forms a 0.22 ± 0.06° angle with the sectioning plane. This angle might have a significant impact on the analysis, as it represents a relative misalignment of approximately 70 µm between two microtissues in first and twelfth well along a line of the HistoBrick array. The tilting angle being different for each HistoBrick sample, the best fitting plane was introduced to enable direct comparison of the microtissues in the seven samples (Fig. 6d). For all HistoBrick samples, independently from the use of the alignment tool, 75% of the microtissues have their center located in average ± 40 µm from the best fitting plane. In other words, 75% of the microtissues have their centers located in a 80 µm thick band in the block.

### Automated sample transfer from 96-well ULA plate

**Figure 7:**
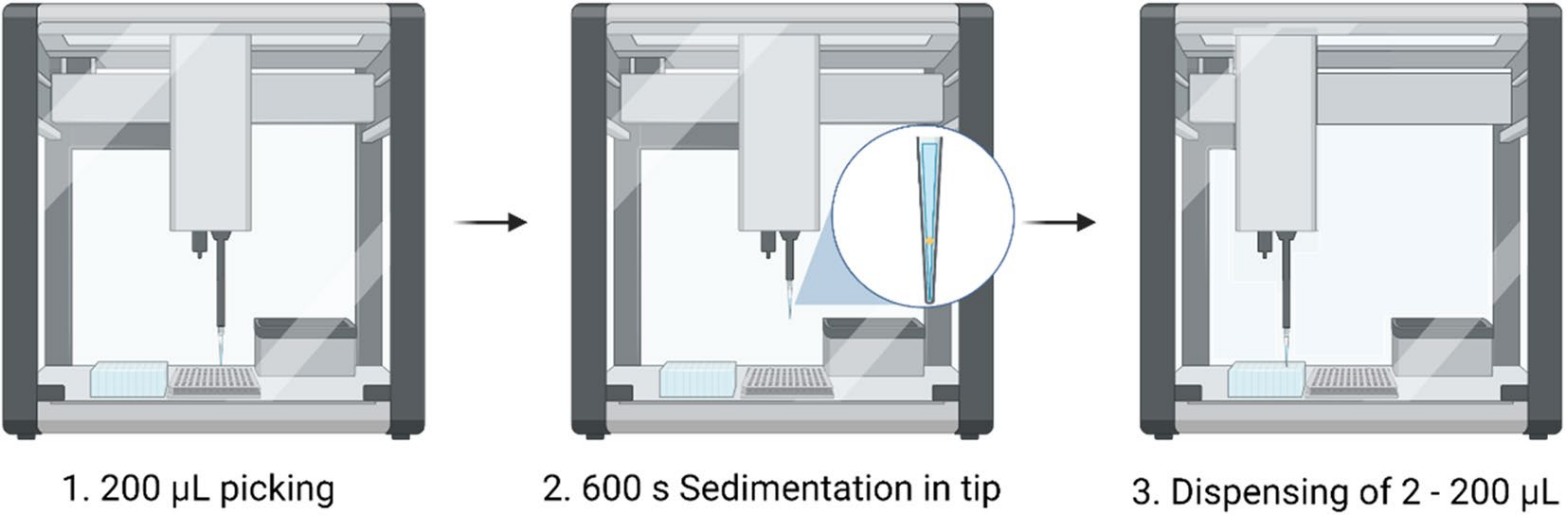
Method development for the transfer of 150–200 µm HepG2 micro-tissues using laboratory automated liquid handler. Created with BioRender.com.

To assess the integration of HistoBrick into an automated workflow, different methods were tested using an automated liquid handler and 96-well ULA plate. Each well contained one HepG2 micro-tissue with 200 µl PBS. Each well on the HistoBrick theoretically enables the collection of 2–10 µl volume. The developed approach consisted of three main steps, as illustrated in Fig. 7. Firstly, the picking of one HepG2 micro-tissue from the ULA plate was successful only when full aspiration of the well volume (200 µl) using 300 µl carbon tips was applied. Any partial volume aspiration tested did not successfully catch the microtissues. Secondly, a pause was implemented so the micro-tissue could sediment at the bottom of the tip. Lastly, the dispensing was investigated in two phases. In the first testing phase, the content of the tip was completely dispensed in a standard 96-well plate. With this approach, 87.5% of HepG2 spheroids were successfully picked-up, transferred and dispensed. This performance depended on coating the dispensing tip (with BSA), the spheroid size and the adherence of the micro-tissue to the bottom of the plate. In the second testing phase, partial volume dispensing was tested to reach the volume range of one HistoBrick’s well (2–10 µl). However, partial jet dispensing of the microtissues within a volume below 20 µl from the 200 µl picked up could not be achieved in a reproducible way. The technical limitations of the hardware, as well as complex method optimisation appeared to be limiting factors, and automated one-to-one transfer of 150–200 µm HepG2 micro-tissues was not possible using this robotic platform.

## Discussion

The application of 3D cell models in the fields of research and personalised medicine is gradually growing as they enable detailed investigations into disease mechanisms, in vitro therapeutic approaches, and therapies planning. The use of patient-derived cells is a major asset, but cell source is limited. In this context, micro-tissue-based research needs to be reliable throughout the entire processing chain. This includes, but is not limited to, micro-tissue production, maturation, testing and end-point analysis. In this context, there is a need for standardisation (which often calls for automation) of the analytical methods that support the development and validation of experimental processes.

This body of work set out to facilitate the histological analysis of micro-tissues by implementing a TMA approach with a focus on cost-efficiency, traceability and compliance with high-throughput laboratory procedures. Previous studies have shown the advantage of using a TMA approach but these reviews were performed on larger specimens (in the range of 500 µm in diameter)^7,8,10,11^. The novel approach presented in this paper, HistoBrick, not only uses small HepG2 micro-tissues (150–200 µm in diameter), it also allows for the precise alignment of the specimens and is compatible with different standard hardware.

HistoBrick would facilitate high-throughput micro-histology by implementing easy-to-use hydrogel labware for the coplanar embedding of 3D cell models, whose design has been made compatible with automation standards. Data collected on HistoBrick demonstrates that it is possible to undertake and gather histological results for up to 96 samples using a smaller selection of 13 slices. The designed silicone mould eases the replication of the mesoscale wells into the agarose-based hydrogel, resulting in a multi-well device that can be stored in humid conditions for at least 14 days before sample loading. The flexibility of the mould and its design with the two openings on both sides of the array enables sufficient bending during unmoulding and helps prevent gel breakage. In addition, HistoBrick’s method is exclusively based on the passive settling and sedimentation of the specimens when they are loaded into the wells and no extra handling steps are required after the micro-tissue is transferred. In terms of performance, HistoBrick shows satisfying compatibility with the whole histological process. Although dimensions of the wells, arrays and samples are affected by the dehydration and paraffin embedding procedures, it is possible to clearly trace the samples as their 2D arrangement is conserved. Moreover, they are aligned on a plane with ~ 80 µm precision range after histological processing. This is particularly relevant as the results were obtained with small HepG2 micro-tissues (150–200 µm in diameter) so that the obtained alignment precision is close to the sample size dispersion. Most recent standardized micro-tissue production platforms are ensuring uniform size amongst an array^19–21^. As the alignment method is based on sedimentation, HistoBrick is particularly addressing the analytical needs of such platforms.

A previous study demonstrated the importance of the positioning of agarose blocks on a microtome when assessing the alignment performance^8^. In this study, an agarose-based TMA was placed in contact with a histology cassette together with agarose, providing support for the sectioning of the gel block, whilst the micro-tissue samples were located at the top of the pillars facing the blade. The HistoBrick blocks were initially dehydrated and embedded with paraffin without any support and the obtained sample alignment plane presented only a 0.2°–0.3° angle with the microtome blade. A customised tool was designed for a complementary paraffin embedding step to help align the block with the blade, which would be useful in the future for less experienced users of the technology. However, it was decided to not investigate the tool’s effectiveness in this instance, as the alignment was excellently arranged by a highly skilled user for this paper. As the alignment of the block with the blade is highly operator dependent, the development of a specialised tool to help standardise this process was still considered as an important step within HistoBrick’s development so it can be easily deployed in the future.

HistoBrick’s wells were designed as a 96-well array on a 1536 grid to comply with the automation standards for liquid handling platforms. If the format and positioning of HistoBrick on the deck layout were successful, limitations were encountered for the automated one-to-one micro-tissue transfer procedure. Ideally, each micro-tissue from the 96-well plate would have been picked up with 2–10 µl of PBS and directly transferred to HistoBrick, then embedded with HistoGel. However, successfully picking single micro-tissues from a round-bottom ULA plate was possible only with the aspiration of the complete 200 µl volume, the micro-tissues being randomly located in the well. After enabling sedimentation of the sample in the tip, partial dispensing of 2–10 µl to the HistoBrick well could not be achieved. The conditions tested in this work aimed at testing one-to-one transfer using a standard liquid handling platform, with micro-tissues isolated in single wells as an input. Those starting conditions were ingeniously considered as a school case, given that large scale standardized micro-tissue production platforms rely on either a pool of micro-tissues in a larger volume (< 1 ml) or isolated micro-tissues in small volume (< 100 µl)^22^. While processes such as pipetting of reagents and medium exchange are standard operations of laboratory automation systems, the transferring of 3D cell models remains challenging. This is mainly due to the precise positioning of the pipette tip above the 3D cell model during aspiration, which requires optical feedback. Without it, successful picking of the micro-tissue is accessible only with a larger volume of liquid. Then, the required partial dispensing of small liquid amounts (< 20 µl) is difficult if not impossible with classical equipment. While bio-printing 3D cell models is an emerging field^23^ and pick and place operations of larger constructs has been demonstrated^24^, little can be found on pick and place operations for the distinct transfer of 3D cell models from one location to another.

In this study, HistoBrick provides a user-friendly, cost-effective and robust method for the embedding of samples with the coplanar alignment of up to 96 micro-tissues. The technology ensures a strong reduction of the number of specimens lost during histological processing thus saving precious biological material. The HistoBrick approach facilitates standardised sample analysis using micro-histology. Efficient use of patient-derived cells is key to promoting the widespread implementation of 3D cell models in research and stratified medicine. The full deployment of the method now relies on the development of liquid handling platforms enabling the efficient transfer of multiple 3D cell models from one labware to another.

## Supplementary Information

Supplementary Information.

## Data Availability

All data described in this manuscript and all details of methods described will be made readily available upon requests made to the corresponding author.
