# Supplementary material for: Coplanar embedding of multiple 3D cell models in hydrogel towards high-throughput micro-histology
Source: Sci Rep. 2022 Jun 15;12:9991. doi: 10.1038/s41598-022-13987-4 (PMC9200833; doi:10.1038/s41598-022-13987-4)
Supplement: Supplementary file 1 — Supplementary Information. [file 41598_2022_13987_MOESM1_ESM.pdf]

Supplemental Methods

Coplanar embedding of multiple 3D cell models in hydrogel towards high-throughput micro-histology

Sarah Heub[1], Fatemeh Navaee[1], Daniel Migliozzi[1], Diane Ledroit[1], Stéphanie Boder-Pasche[1], Jonas Goldowsky[1], Emilie Vuille-Dit-Bille[1], Joëlle Hofer[2], Carine Gaiser[2], Vincent Revol[1], Laura Suter-Dick[2,3], Gilles Weder[1]*

[1] CSEM SA, Jaquet-Droz 1, 2002 Neuchâtel, Switzerland
[2] School of Life Sciences, University of Applied Sciences and Arts Northwestern Switzerland, 4132 Muttenz, Switzerland
[3] Swiss Centre for Applied Human Toxicology (SCAHT), 4001 Basel, Switzerland

*e-mail: sarah.heub@csem.ch

S1 – HepG2 micro-tissue culture and fixation

For this work, human hepatocellular carcinoma (HepG2) (HB-8065™, ATCC) cells were cultured in Dulbecco's Modified Eagle Medium (DMEM) supplemented with 10 % fetal bovine serum (FBS) and 1 % penicillin-streptomycin. When the cells reached 80 % confluency, they were passaged. To culture the HepG2 micro-tissues, the cells were trypsinised from a 75 cm^2 flask and then counted using trypan blue solution to calculate the percentage of viability. Sphericalplate 5D platform technology (SP5D, Kugelmeiers Ltd.) was used to culture the HepG2 micro-tissues, with 200 cells per micro-well (i.e., 150,000 cells per well, with each well containing 750 micro-wells) (Figure S1.). The medium was changed every other day. The HepG2 micro-tissues were harvested after six days of culturing and fixed with 4 %

paraformaldehyde (PFA) for 20 minutes at room temperature. After 20 minutes, the PFA was replaced with phosphate-buffered saline (PBS). The fixed micro-tissues can be stored in the fridge for a month in a larger vial (15 ml Falcon® Tube).

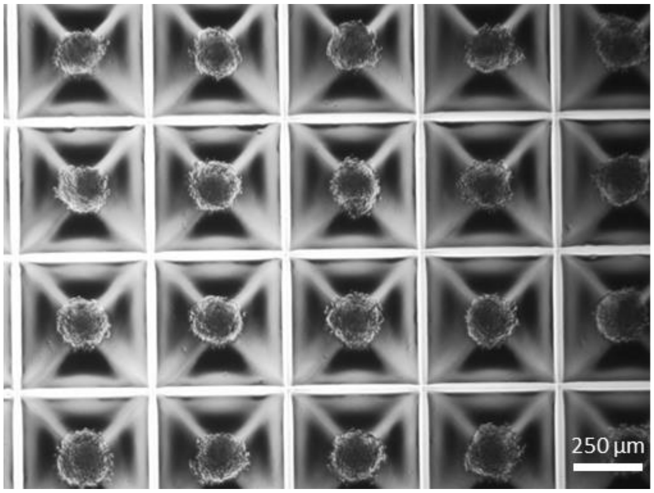

Figure S1. HepG2 spheroids formed in Kugelmeiers' Sphericalplate 5D platform technology.

S2 - Design and fabrication of a slicing tool

When designing HistoBrick, the authors behind this paper considered two areas on the outer sides of the wells that would help future users handle the block. However, after preparing the HistoBrick using the silicone mould, it was found to be necessary to remove the two outer side sections of the HistoBrick to fit a histology cassette. For this reason, a customised tool was designed with dimensions that match that of the HistoBrick. Two blades were placed at the border of the outer sides, which trim the block accordingly (Figure S2.).

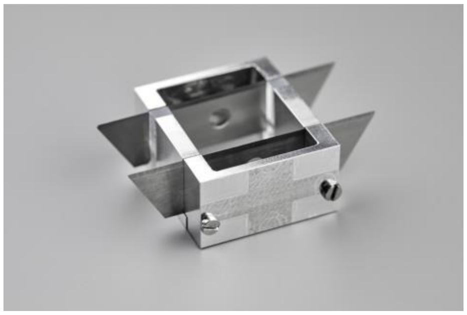

Figure S2. Slicing tool. Two metallic blades were clamped in a metallic frame matching the dimensions of the HistoBrick.

S3 - Design and fabrication of an alignment tool

Mounting the paraffin-embedded sample block is a manual, operator-dependent procedure. As is common practice, paraffin embedding of gel blocks is performed by placing the sample in a chamber,

dehydrating, clearing and filling it with liquid paraffin, where it then hardens. The user then takes the risk of placing the obtained block on the microtome with a tilted angle, as there is no guiding structure available to prevent misalignment. The procedure thus requires highly skilled personnel to precisely align the sample with the microtome blades and to ensure that subsequent sections will be obtained in the plane of the embedded micro-tissues. A tool (Figure S3.) was designed to facilitate the positioning of the block on the microtome. It guides and guarantees the positioning of the section plane parallel to the bottom surface of the HistoBrick, i.e., to the embedded micro-tissue plane. The method uses a custom chamber and a histology cassette that provides support with a flat surface. Firstly, the HistoBrick containing the micro-tissues undergoes dehydration, clearing and paraffin embedding. Then, an additional embedding step is performed. The working procedure is as follows: a histology cassette is positioned on the base of the alignment tool. Then, the paraffin-embedded HistoBrick is placed with its flat surface facing the histology cassette. The metallic alignment tool is then adjoined. The components are then filled with melted paraffin and allowed to cool down at room temperature until solidification is achieved. At this point, the flat surface of the HistoBrick can be laid parallel to the histology cassette surface, and it can be easily mounted parallel to the microtome blade.

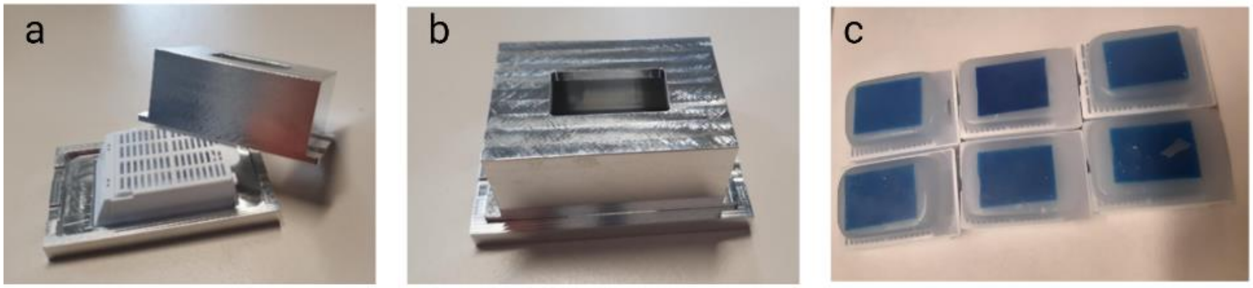

Figure S3. Alignment tool. (**a**) The histology cassette is placed in the alignment tool, (**b**) The HistoBrick is positioned on top of the cassette and the alignment tool is adjoined. Once closed, the tool enables a second step of paraffin embedding, providing rigid and planar support for the processed gel block, (**c**) The paraffin-embedded blocks. The HistoBrick is colored with blue histology dye for having a better contrast.
